# Case report: Aqueous and Vitreous amino-acid concentrations in a patient with maple syrup urine disease operated on rhegmatogenous retinal detachment

**DOI:** 10.1186/s12886-016-0349-3

**Published:** 2016-10-03

**Authors:** Menelaos G. Kanakis, Helen Michelakakis, Petros Petrou, Chrysanthi Koutsandrea, Ilias Georgalas

**Affiliations:** 1 1st Department of Ophthalmology, “G. Gennimatas” Hospital of Athens, National and Kapodistrian University of Athens, Mesogeion 154, Athens, 11527 Greece; 2 Department Enzymology and Cellular Function, Institute of Child Health, 7 Fokidos street, Athens, 11526 Greece

**Keywords:** Case report, Maple syrup urine disease, Retinal detachment, Amino acid concentration, Vitreous

## Abstract

**Background:**

Maple syrup urine disease (MSUD) is a rare metabolic disorder, affecting the metabolism of branched chain amino-acids (Valine, Leukine, Isoleukine). We present a rare case of rhegmatogenous retinal detachment (RRD) in a MSUD patient.

**Case presentation:**

We performed amino acid analysis of aqueous humour, vitreous and serum samples obtained during surgery from a 24 year old female MSUD patient successfully operated on RRD.

Serum values for a-amino-butyric acid, valine, isoleucine, leucine, tyrosine, phenylalanine, ornithine and histidine were low, while values for citrulline, methionine and lysine were borderline low, all attributed to the patient’s special diet. Serum glutamate was above normal, probably due to the breakdown of glutamine to glutamate.

In the aqueous and vitreous the amino acids implicated in MSUD (Valine, Leukine Isoleukine), were within normal range. Glutamate was absent in the vitreous and presented low levels in the aqueous. Glutamate has been reported to play an important role in retinal damage. Elevated glutamate levels have been reported in vitreous specimens from patients subjected to vitrectomy or buckling surgery for RRD. In MSUD, glutamate has been implicated in the pathogenesis of brain damage. Low levels of glutamate have been observed in the cerebellum of experimental MSUD animals, as well as postmortem brain tissue from a child that died of leucine intoxication. The reduction was attributed to the elevation of a-ketoisocaproic which reverses the net direction of nitrogen flow. It could be argued that this could impact on amino acid concentration in aqueous and vitreous fluids.

**Conclusions:**

Although no definite conclusions can be drawn by this extremely rare case, the low vitreous and aqueous levels of Glutamate is an interesting finding. Further studies are needed to provide a better insight in the role of amino acids as neurotransmitters in the human eye in health and disease.

**Electronic supplementary material:**

The online version of this article (doi:10.1186/s12886-016-0349-3) contains supplementary material, which is available to authorized users.

## Background

MSUD is a rare (1:200,000) genetic metabolic disease, caused by a defect in branched chain ketoacid dehydrogenase (BCKA), leading to elevated concentrations of the branched-chain amino acids leucine, isoleucine, and valine [1]. This enzyme is a supra enzyme complex consisting of multiple copies of four distinct subunits and is located in the inner wall of the mitochondrion. The disease has autosomal-recessive inheritance. Ocular complications of untreated disease or late diagnosis include optic atrophy, nystagmus, ophthalmoplegia, strabismus, and cortical blindness [2].

## Case presentation

A 24 year old Caucasian female was admitted to our hospital when diagnosed with Rhegmatogenous Retinal Detachment (RRD), on her left eye. The patient also suffers from maple syrup urine disease (MSUD), for which she has been maintained on a special diet and thiamine supplements, autismus (under treatment with quetiapine, oxcarbazepine and fluvoxamine), and ulcerative colitis (under treatment with mesalamine) (Table 1) (Additional file 1).

**Table 1:** Timeline (following the CARE Guidelines)

• Diagnosed with the aid of newborn screening and commenced on early dietary restriction	1991
• Repeated episodes of metabolic decompensation, usually in the context of catabolic stress associated with non-specific illness, with subsequent brain injury	1991–2007
• Diagnosed with ulcerative colitis	2012
• Diagnosed with Rhegmatogenous Retinal Detachment	2015
• Operated on successfully under general anesthesia, with 23G Vitrectomy	
• Follow up (4 months)	2016

Dilated fundus examination revealed an inferior retinal detachment (3 – 8^th^ h), macula off. The detachment had been discovered 12 days ago and there were not any signs of Proliferative vitreoretinopathy (PVR). The patient was operated on successfully under general anesthesia, with 23G Vitrectomy (Constellation Vitrectomy System, Alcon Laboratories, Texas, USA), and is under follow up for the last four months. Apart from the successfully treated retinal detachment, slit lamp biomicroscopy and fundus examination were unremarkable on admission and during the follow up period.

Informed consent of the custodian (mother) was obtained, since the patient is mentally retarded due to MSUD. Undiluted vitrectomy samples (approximately 0.5 ml) were obtained by a conventional three-port closed vitrectomy technique, by manual suction at the beginning of the vitrectomy, before opening the infusion line of Balanced Salt Solution (BSS, Alcon Laboratories, Texas, USA), as described earlier by Diederen et al. [3]. Aqueous humour was obtained by anterior chamber paracentesis with 30G insulin syringe (approximately 100 μl). The serum sample was also obtained at the same time. The aqueous and vitreous samples were transferred to Eppendorf tubes and stored frozen, until the time of amino acid analysis, performed at the Institute of Child Health, Department of Enzymology.

Analysis was performed by ion exchange chromatography using the Biochrom 30 Amino Acid Analyser.

The results of the amino-acid analysis are presented in the Table 2: Amino acid concentration in aqueous, vitreous and serum, along with the findings of eight other studies [3–10] on amino-acid concentration in ocular fluids. This was regarded necessary, as normative data are scarce (Table 2).

**Table 2:** Amino acid concentration in aqueous, vitreous and serum

Amino acid	Aqueous (μM)				Vitreous (μM)									Serum (μM)	
	MSUD	Wuu et al.^a^	Durham^b^	Wakabayashi et al.^c^	MSUD	Bertram et al.^d^		Diederen et al.^e^		Durham^b^	Honkanen et al.^f^	Asensio et al.^g^	Yalcinbayir et al.^h^	MSUD	LAB
		Aqueous control	Aqueous control	Aqueous control		Vitreous RRD	Vitreous control	Vitreous RRD	Vitreous control	Vitreous control	Vitreous control	Vitreous RD,MH,ERM	Vitreous Control		Normal
Pro	45	16.9 ± 15.4	16 ± 5.3		U					4 ± 5.6				101	97–297
Tau	46	29.3 ± 12	39.1 ± 11.3	22.25 ± 8.36	21			26.0 ± 7.8	22.6 ± 6.6	28.8 ± 5		11.9 ± 1.3	79(26–146)	61	27–95
Asp	3	13.4 ± 9.1	1 ± 1.5	1.81 ± 1.04	3	U	1.1 ± 0.8	6.7 ± 2.8	6.6 ± 2.5	1.7 ± 1.5	1.4 ± 1.0	3.7 ± 4.1	47(6–70)	5	2–9
Thr	158	106.7 ± 18.9	152 ± 41.4		31					73.6 ± 10.3	85.5 ± 28.4	61.8 ± 3.6	385(77–519)	130	92–180
Ser	131	111.2 ± 45.3	179 ± 38.1		89					106 ± 24.4	103.9 ± 24.4	79.2 ± 19.5	501(96–733)	99	89–165
Asn	78	27.3 ± 11.1	49.2 ± 11.5		39					24.3 ± 4.3	35.8 ± 11.6	19.5 ± 8.1		80	32–92
Glu	6	39.5 ± 18.8	12 ± 3.4	2.98 ± 1.12	U	13.4 ± 11.9	1.7 ± 0.8	16.6 ± 5.6	13.1 ± 5.2	14.5 ± 11	5.2 ± 2.3	3.8 ± 5.1	20(10–82)	96	6–62
Gln	658	95.9 ± 68.9	717 ± 134		627						1192.9 ± 404.4		3386(714–4698)	599	466–798
Gly	71	7 ± 3.2	16.7 ± 3.5		5	U	4 ± 2.7	42.5 ± 32.0	49.4 ± 36.3	8.7 ± 2.6	8.5 ± 2.5	10.2 ± 6.2	56(15–196)	256	147–299
Ala	227	182.7 ± 91.4	294 ± 66.1	105.98 ± 9.88	85	U	88.4 ± 35			126 ± 15.6	159.5 ± 54.9	31.8 ± 10.6	896(125–1842)	241	146–494
Cit	6	3.8 ± 6.6	9.8 ± 4.8		6					8.3 ± 1.6			9(5–27)	18	19–47
Abu	10	10.6 ± 5.7	31.4 ± 7.8		U					14.4 ± 1.7				9	15–35
Val	146	281.2 ± 180.6	388 ± 84.5		67	U	112 ± 4.4			174 ± 41.3		87.5 ± 6.5	909(140–1277)	95	179–335
Cys	9	7.9 ± 1.7	11.2 ± 3.5		4					6.5 ± 6				42	24–54
Met	23	18.8 ± 8.6	44.4 ± 9.9		13					24.7 ± 5	22.3 ± 8.1	13.2 ± 3.2	84(12–133)	13	13–37
Ile	52	72.2 ± 26.2	79.8 ± 6.9		32	U	28.3 ± 12.0			37.6 ± 3.4	37.9 ± 11.6	32.8 ± 5.1	220(39–317)	27	46–90
Leu	132	169.5 ± 72.2	192 ± 39.6		72	U	63.5 ± 21.9			96.1 ± 20.5	89.7 ± 28.4	76.5 ± 5.3	584(93–737)	88	113–205
Tyr	58	79.2 ± 37.6	123 ± 16.5		32	U	31.9 ± 3.7			51.8 ± 12.3	58.2 ± 18.8	41.1 ± 6.3	365(58–399)	29	37–77
Phe	55	97.7 ± 30.3	119 ± 14		28	U	31.7 ± 11			56.8 ± 13.6	44.4 ± 14.2	43.3 ± 1.9	356(66–469)	29	46–74
Orn	22	15 ± 3.4	25.3 ± 4.6		15					16 ± 1.1		13.6 ± 0.9		42	55–135
Lys	168	110.3 ± 36.5	155 ± 23.5		114					105 ± 14.4	115.4 ± 33.7	87.6 ± 4.6		137	135–243
His	60	47.6 ± 9.5	77.9 ± 12.7		29					40.7 ± 13.8	38.4 ± 10.0			62	72–108
Trp	36	U	31.9 ± 10		U					18.3 ± 7		7.4 ± 1.2		24	n/a
Arg	100	29.2 ± 13	133 ± 26		66	U	52.7 ± 46.0			71.9 ± 10.2		62.3 ± 10.5		46	28–96

*MSUD* maple syrup urine disease-i.e., our patient, *μM* μmol/l, *U* unmeasurable; empty cells refer to not available data

*MH* macular hole, *ERM* epiretinal membrane, *a.c.* anterior chamber, *RRD* rhegmatogenous retinal detachment, *TRD* tractional retinal detachment, *PPV* pars plana vitrectomy, *LAB(normal)* normal reference values provided by the laboratory that performed the amino acid analysis

Data regarding previous studies

^a^ Wuu et al. [4]: Controls: Aqueous: *n* = 3 (ophthalmological emergency operations - Chinese population)

^b^Durham [5]: Controls: Aqueous: normal eyes, a.c. paracentesis (*n* = 9, mean age 28y (2.5–77). Vitreous: procedure with vitreous loss (*n* = 3, Marfan/cataract/enucleation, age = 40/21/35 y)

^c^ Wakabayashi et al. [6]: Controls: cataract without other ocular disease (*n* = 9, age 66.1 ± 12.8 y.)

^d^ Bertram et al. [7]: PPV for RRD (*n* = 5, age =63.8 ± 15.5 y., duration 52.4 ± 65.4 days). Controls: vitrectomy for MH (*n* = 7) or ERM (*n* = 3) (age =66.1 ± 12.1 y.)

^e^ Diederen et al. [3]: PPV for primary retinal detachment (*n* = 114, age = 58.2 ± 15.1 y., duration 43.6 ± 72.1 days). Controls: PPV for MH or ERM (*n* = 52, age = 51.3 ± 14.0 y.)

^f^ Honkanen et al. [8]: Controls: PPV for MH (*n* = 10 [2 RRD]), ERM(*n* = 6[2 TRD]), MH and ERM (*n* = 1). Total number of eyes: (*n* = 17, age = 68 ± 14 y.)

^g^ Asensio et al. [9]: 64 eyes with RRD *n* = (45), MH (*n* = 5) and ERM (*n* = 14). Age = 67.7 ± 14.2 y. [No statistically significant difference neither between groups, nor regarding age]

^h^ Yalcinbayir et al. [10]: Controls: PPV for MH(*n* = 6), subluxated lens (*n* = 1). Age = 67.3 ± 3.8 y

We preferred to compare to normal control values as well as values obtained from patients with RRD from vitreous fluid during vitrectomy when possible, although data from subretinal fluid (SRF) obtained during buckling surgery, were available. As for the aqueous humour, only values from normal controls were available. Normative data for serum levels were provided by the laboratory that performed the analysis. All values are presented as μmol/l (μM). In the table we adopted the standard abbreviations for amino acids. Data regarding the patients and control groups in the aforementioned papers are presented in brief.

The patient presented with low serum levels of several amino-acids. Values for a-amino-butyric acid (10 μM), valine (146 μM), isoleucine (52 μM), leucine (132 μM), tyrosine (58 μM), phenylalanine (55 μM), ornithine (22 μM) and histidine (60 μM) were low and values for citrulline (6 μM), methionine (23 μM) and lysine (168 μM), borderline low. Only values of Glutamic acid (6 μM) were detected to be above normal values in the serum. Amino-acid concentration in the aqueous was higher than that of the vitreous, apart from glutamine (658 μM and 627 μM respectively), aspartic acid (3 μM and 3 μM respectively), and citrulline (6 μM and 6 μM respectively). All three amino acids implicated in the metabolic disorder (Valine, Leucine, Isoleucine), were within the reported normal ranges in the aqueous (146 μM, 132 μM, 52 μM respectively) and vitreous (67 μM, 72 μM, 32 μM respectively). Glutamate levels in were unmeasurable in the vitreous and low in the aqueous (6 μM), while Taurine levels were found to be close to normal limits for aqueous (46 μM) and vitreous (21 μM).

The low serum levels of several amino-acids in the serum were probably due to the fact that the patient has been maintained on a strict special diet. However, values of Glutamic acid were detected to be above normal in the serum. This can be explained, since glutamine is converted to glutamate on storage, and the sum of glutamine + glutamate is well within the normal range. It could be argued that the increased level of glutamate observed most probably reflects the breakdown of glutamine during storage.

Notably there is not an established ratio of serum to aqueous or vitreous amino-acid concentration [11, 12]. On the contrary there has been reported that amino-acid concentration in the aqueous is higher than that of the vitreous, apart from glutamine [5], which seem to be confirmed by the nearly identical concentrations in our specimens (658 μM and 627 μM respectively). The serum concentration of glutamine also lies within the same range (599 μM) [5]. In our specimens nearly identical amino-acid concentrations between aqueous and vitreous have also been found for aspartic acid and citrulline [5].

All three amino acids implicated in the metabolic disorder (Valine, Leucine, Isoleucine), were within the reported normal ranges in the aqueous and vitreous. This is not that strange, as it has been reported that CSF amino acid concentrations do not reflect serum concentration, and blood–brain barrier has its equivalent in the eye in the form of blood-aqueous and blood-retina barriers [13].

On the other hand, presumably important factors that determine the concentration of amino acids in aqueous and vitreous are not yet clear. The active efflux of amino acids in the aqueous [14, 15], combined with the turnover rate of the aqueous and the sink effect of the vitreous itself [15], as well as the variability of amino acid concentration in the normal vitreous comprise a dynamic equilibrium system.

Another interesting aspect of our results is the absence of glutamate in the vitreous, and the low levels in the aqueous. Glutamate has been reported to play an important role in retinal damage, and elevated glutamate levels have been reported in vitreous specimens from patients subjected to vitrectomy for RRD. [3, 7] In RRD patients treated with scleral buckle, elevated levels of glutamate in Subretinal fluid (SRF) have also been reported. In these patients SRF glutamate levels seem to rise in parallel with the duration of the detachment [10]. Elevated glutamate levels in the aqueous have also been reported in cases of retinal artery occlusion [6], and in glaucoma patients [8].

Glutamate is the main excitatory neurotransmitter in the central nervous system (CNS) and the retina [16], and is constantly released from the synapses of photoreceptors but also bipolar and amacrine cells, in a Ca^+2^-dependent manner. It also seems that there is a Ca^+2^-independent mechanism under stress conditions (hypoxia), by reversal of the plasma membrane uptake carrier. Glutamate is normally removed from the extracellular space by the glutamate/aspartate transporter system, by neuronal and glial cells. Failure to remove glutamate from the extracellular space, causes an increase in glutamate concentration and leads to overstimulation of *N-*methyl- D-aspartate receptor, leading to Ca^+2^ influx into cells. The increased intracellular Ca^+2^ leads to the production of nitric oxide (NO), that interacts with oxygen to form extremely toxic free radicals resulting in retinal neuronal death [17, 18]. Regarding retinal detachment, an acute efflux of neuronal glutamate, caused by injury-mediated acute depolarization and release of glutamate vesicles may also trigger the above mechanism and lead to excitotoxic cell death in the detached retina [18, 19].

In MSUD glutamate has been implicated in the pathogenesis of brain damage.

Low levels of glutamate have been observed in the cerebellum of experimental MSUD animals [20, 21] as well as postmortem brain tissue from a child that died of leucine intoxication [22]. The reduction was suggested to result from the elevation of a ketoisocaproic which reverses the net direction of nitrogen flow. It could be argued that this could impact on amino acid concentration in aqueous and vitreous fluids. Although a correlation was found between increased vitreous glutamate and a lower pre-operative visual acuity, this was not the case regarding visual outcome.

Diederen et al. [3] detected that apart from glutamate, taurine levels were also increased in RRD. In our patient taurine levels were found to be within normal limits. Taurine is one of the most abundant amino acids in the brain and retina, although it is one of the few amino acids not incorporated into proteins. In the retina, taurine is critical for photoreceptor development and acts as a cytoprotectant against stress-related neuronal damage [23].

## Conclusions

It is obvious that no definite conclusions regarding amino acid concentration in aqueous and vitreous of MSUD patients with RRD can be drawn by this case. We present our data not only because this is a rare case of RRD in a MSUD patient (no other case has been reported till now according to our best knowledge), but also to draw attention to rare diseases and metabolic disorders with ophthalmological complications that, if studied adequately, may provide useful data for the patients care, as well as a better insight in the role of amino acids as neurotransmitters in the human eye.

## Additional file

Additional file 1: Care flow diagram. (DOCX 54 kb)

## Abbreviations

a.c.: Anterior chamber
Abu: α-Aminobutyric acid
Ala: Alanine
Arg: Arginine
Asn: Asparagine
Asp: Aspartic acid (Aspartate)
BCKA: Branched chain ketoacid dehydrogenase
BSS: Balanced salt solution
Cit: Citrulline
Cys: Cysteine
ERM: Epiretinal membrane
Gln: Glutamine
Glu: Glutamate
Gly: Glycine
His: Histidine
Ile: Isoleucine
LAB: The laboratory that performed the amino acid analysis
Leu: Leucine
Lys: Lysine
Met: Methionine
MH: Macular hole
MSUD: Maple syrup urine disease
NO: Nitric oxide
Orn: Ornithine
Phe: Phenylalanine
PPV: Pars plana vitrectomy
Pro: Proline
RRD: Rhegmatogenous retinal detachment
Ser: Serine
SRF: Subretinal fluid
Tau: Taurine
Thr: Threonine
TRD: Tractional retinal detachment
Trp: Tryptophan
Tyr: Tyrosine
Val: Valine
μM: μmol/l
