# Supplementary figures and images for: Case report: Aqueous and Vitreous amino-acid concentrations in a patient with maple syrup urine disease operated on rhegmatogenous retinal detachment
Source: BMC Ophthalmol. 2016 Oct 3;16:170. doi: 10.1186/s12886-016-0349-3 (PMC5048685; doi:10.1186/s12886-016-0349-3)

**CARE FLOW DIAGRAM**


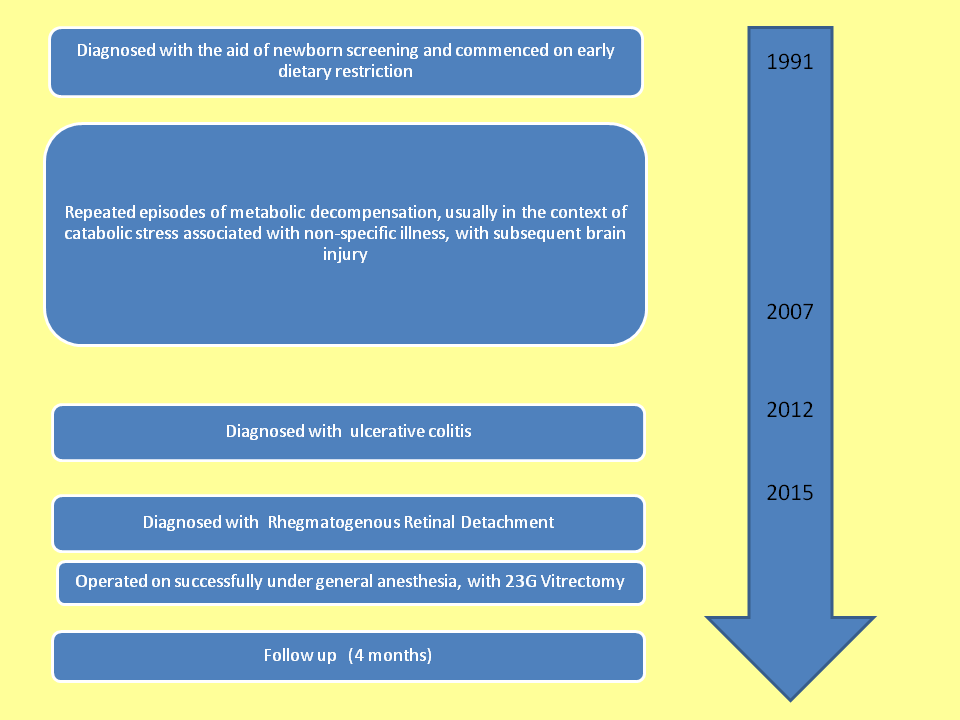

Supplement: Additional file 1: — Care flow diagram. (DOCX 54 kb) [file 12886_2016_349_MOESM1_ESM.docx]
